# Association of laboratory markers and cerebral blood flow among sickle cell anemia children

**DOI:** 10.3389/fped.2022.914466

**Published:** 2022-08-26

**Authors:** Corynne Stéphanie Ahouéfa Adanho, Sètondji Cocou Modeste Alexandre Yahouédéhou, Sânzio Silva Santana, Camilo Vieira, Rayra Pereira Santiago, Jeanne Machado de Santana, Thassila Nogueira Pitanga, Milena Magalhães Aleluia, Vítor Valério Maffili, Ivana Paula Ribeiro Leite, Dalila Luciola Zanette, Isa Menezes Lyra, Marilda Souza Goncalves

**Affiliations:** ^1^Laboratório de Investigação em Genética e Hematologia Translacional, Instituto Gonçalo Moniz, Salvador, Bahia, Brazil; ^2^Laboratório de Pesquisa em Anemia, Departamento de Análises Clínicas, Faculdade de Farmácia, Universidade Federal da Bahia, Salvador, Bahia, Brazil; ^3^Faculdade de Biomedicina, Universidade Católica do Salvador, Salvador, Bahia, Brazil; ^4^Ambulatório Pediátrico de Doença Cerebrovascular, Hospital Universitário Professor Edgard Santos, Universidade Federal da Bahia, Salvador, Bahia, Brazil; ^5^Departamento de Ciência Biológicas, Universidade Estadual de Santa Cruz, Ilhéus, Bahia, Brazil; ^6^Serviço de Pediatria, Hospital Universitário Professor Edgard Santos, Universidade Federal da Bahia, Salvador, Bahia, Brazil; ^7^Ambulatório, Fundação de Hematologia e Hemoterapia da Bahia, Salvador, Bahia, Brazil; ^8^Curso de Medicina, Escola de Ciências da Saúde e Bem-Estar, Universidade Salvador, Salvador, Bahia, Brazil

**Keywords:** sickle cell anemia, stroke, Transcranial Doppler, laboratory parameters, TAMMV

## Abstract

**Background:**

Stroke is one of the highest complications of sickle-cell anemia (SCA). The Transcranial Doppler (TCD) has been adopted worldwide as a gold standard method for detecting alterations in the blood velocity in cerebral arteries. In this study, we investigated the association between laboratory parameters and increased cerebral blood flow velocity in Brazilian SCA pediatric patients.

**Methods:**

The study included 159 pediatric patients with SCA, submitted to TCD velocity screening, and the time-averaged maximum mean velocity (TAMMV) was determined in the middle cerebral artery (MCA), anterior cerebral artery (ACA), and distal intracranial internal carotid artery (ICA). We compared cerebral blood flow in patients stratified by the following: TCD1—defined as normal, with TAMMV inferior to 170 cm/s; TCD2—conditional, with TAMMV above 170 cm/s, but less than 199 cm/s; TCD3—altered, with TAMMV greater than or equal to 200 cm/s.

**Results:**

TAMMV was negatively correlated with age and weight (*p* < 0.05). Moreover, TAMMV was associated or correlated with reductions in HbF, RBC, hemoglobin, hematocrit, HDL, and haptoglobin and, increases in MCV, MCH, RDW, reticulocytes, WBC, lymphocytes, monocytes, eosinophils, total and indirect bilirubin, LDH, AST, ALT, glucose, ferritin, and AAT (*p* < 0.05).

**Conclusion:**

The current study highlights the importance of the investigation of hemolytic and inflammatory biomarkers for monitoring the clinical outcome of SCA pediatric patients, to avoid acute or chronic stroke. Moreover, glucose and HDL-C appear useful for predicting higher TAMMV.

## Introduction

Sickle-cell anemia (SCA) has an overly complex range of clinical manifestations that depend on many biological and genetic factors, and some are not well-understood. The intravascular hemolysis and chronic anemia that occur in patients SCA play an important role in the disease pathophysiology and clinical event variability. Cerebral vascular accidents (CVA), asymptomatic or symptomatic events, are one of the impairing consequences of SCA and are associated with neurocognitive dysfunction (1–3). Stroke physiopathology can be explained by a microvascular or macrovascular occlusion (4–6).

It frequently occurs during the five (5) first years of life (7), and affects nearly 11% of SCA patients under 20 years of age and, 20–30% will have a silent infarction (4, 8, 9). The same study from the Cooperative Study of Sickle Cell Disease demonstrated that the annual incidence of the first stroke for those with SCA was 0.6% per patient/year with the highest rate of 1.02% per patient/year seen in the group aged 2–5 years. The cumulative risk of stroke was 11% in individuals with age lower than or equal to 20 years, increasing to 24% in those from 20 to 45 years old (8, 9). Many studies have attracted attention to the endothelial dysfunction associated with intravascular hemolysis as an important event in the pathophysiology of stroke in SCA (10–13). Kato et al. (11) made a point about how the hemolytic profile affects the blood flow velocity in the large cerebral vessel, being a risk of stroke event occurrence (11). For decades, stroke has been studied and it happens much progress in the knowledge, especially according to the prevention and early treatment, based on transfusion or/and hydroxyurea (HU) (4, 14, 15). Adams (16) demonstrated that Transcranial Doppler (TCD) velocities can predict stroke risk by detecting that patients with elevated blood flow are more susceptible to a CVA event (9, 16, 17). The TCD has been adopted worldwide as a gold standard method for detecting alteration in the blood velocity in cerebral arteries. Based on the evidence, blood velocity greater than or equal to 200 cm/s is considered a stroke risk condition (17, 18). Currently, some predictors are considered classic markers, such as severe anemia, leukocytosis, nocturnal hypoxemia, and genetic factors (7, 19, 20). Several studies have been conducted to identify risk factors associated with CVA in individuals with SCA (21–24). In this study, we investigated the association between laboratory markers and cerebral blood flow velocity in SCA pediatric patients.

## Materials and methods

### Subjects and exclusion criteria

**FIGURE 1:**
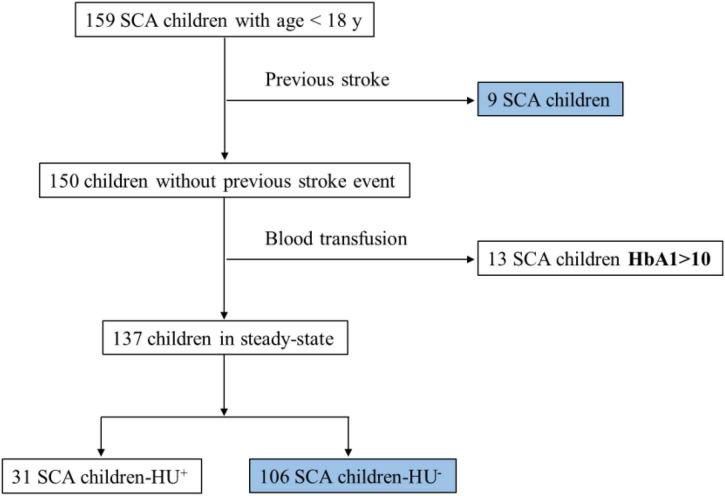
Study design. SCA, sickle cell anemia; HU^+^, with HU treatment; HU-, without HU treatment; y, years.

A cross-sectional study was performed between June 2014 and July 2016, and initially included 159 SCA (HbSS) pediatric patients, all seen at the Professor Hosannah de Oliveira Pediatric Center, part of the Professor Edgard Santos University Hospital Complex (CPPHO/HUPES), located in Salvador, and at the Sickle Cell Disease Reference Center of Itabuna. Both the cities are in the state of Bahia, Brazil. We excluded from the study 09 patients who reported a previous stroke event and 13 who were submitted to blood transfusion a few times before enrollment in the study. Of the 137 patients in steady-state (i.e., absence of acute crisis and no use of blood transfusion in the 3 months before blood collection), 31 (22.63%) patients reported HU treatment and were also excluded, due to the association of its use with alterations in laboratory markers (Supplementary Table 1). One hundred and six (106) patients were then considered for the analyses performed in this study, all seen without active infection or inflammatory diseases (Figure 1).

### Ethical aspects

This study received approval from the Institutional Review Board of the HUPES (protocol number: 287.768/2013) and was conducted in compliance with the Declaration of Helsinki (1964) and its subsequent amendments. The patient’s legal guardians provided a signed term of informed consent before enrollment in the study.

### Transcranial Doppler ultrasonography

TCD were examined in all children by the same-trained professional and using the same equipment. Time-averaged maximum mean velocity (TAMMV) was assessed using a 2 MHz probe connected to a Doppler-Box™ X sonography system (Compumedics Germany GmbH, Singen, Hohentwiel, Germany). The TAMMV was recorded in the middle cerebral arteries, anterior cerebral, and distal intracranial internal carotid arteries. Accordingly, 03 outcomes were considered, TCD1–defined as normal, TAMMV inferior to 170 cm/s; TCD2–conditional, with a TAMMV above 170 cm/s and less than 199 cm/s; TCD3—altered, TAMMV greater than or equal to 200 cm/s (9). We did not assess brain imaging during the follow-up and none of the participants of the study had a stroke or started new therapies.

### Hematological and biochemical parameters

Blood samples were collected by venipuncture, after 12 h of fasting, in an ethylene diamine tetraacetic acid (EDTA) tube for hematological profiling and a dry tube without anticoagulant for biochemical analysis, on the same day on which TCD was performed. Hematological data was obtained using a CELL-DYN Ruby Hematology Analyzer (Abbott Diagnostics, Lake Forest, Illinois, United States), and hemoglobin profiling was performed by high-performance liquid chromatography using an HPLC/Variant II Hemoglobin Testing System (Bio-Rad, Hercules, California, United States). Biochemical analyses, such as lipid and profiles, total proteins and fractions, total bilirubin and fractions, lactate dehydrogenase (LDH), alanine transaminase (ALT), aspartate transaminase (AST), and iron serum levels, were investigated using a spectrophotometer autoanalyzer (Biosystems SA, Barcelona, Spain). Ferritin quantification was performed using an Access 2 Immunochemistry System (Beckman Coulter, Inc., Fullerton, CA, United States). C-reactive protein (C-RP) and alpha-1 antitrypsin (AAT) measurements were performed using an Immage 800 system (Beckman Coulter, Fullerton, CA, United States).

### Statistical analysis

A quantitative variable distribution was developed using the Shapiro-Wilk test. The mean values between the two groups were compared using the Mann-Whitney *U* test for variables with a non-normal distribution or the independent *t*-test for variables with a normal distribution. Regarding the comparisons of the mean values between more than two groups, the Kruskal-Wallis or ANOVA was used for variables with a non-normal or normal distribution, respectively. Frequencies of qualitative variables were also calculated. The Spearman correlation test was performed to investigate the relationship between the two quantitative variables. Statistical analyses were performed using Epi Info 7.0, GraphPad Prism 6.0, and SPSS 17.0. *P*-values less than 0.05 were considered statistically significant. Data were expressed as mean ± standard deviation (M ± SD), fifty percentiles (minimum-maximum), and number or frequency where appropriate.

## Results

### Demographic and laboratory parameters of the sickle-cell anemia children according to Transcranial Doppler

The analysis of demographic data showed that 45 (42.86%) patients were female. The median age was 6.5 ± 3.6 years (range: 2–17 years), 46 (43.39%) patients had ages ranging between 2 and 5 years. Table 1 and Supplementary Figure 1 present the results of laboratory parameters according to TCD. We observed significantly lower TAMMV values in TCD1 patients compared with those with TCD2 or TCD3. The analysis of biomarkers related to hemolysis and hepatic injury showed that patients with TCD3 had significantly reduced RBC, and hematocrit, as well as increased MCV, MCH, and AST compared with those with TCD1 (*p* < 0.05). Regarding leukocyte count and inflammatory profiles, increased counts of WBC, lymphocytes, monocytes, and AAT levels were observed in the TCD3 group compared with those with TCD1 (*p* < 0.05). We also observed an increase in glucose levels in the TCD3 group vs. TCD1 group (*p* < 0.05).

**TABLE 1:** Laboratory parameters of children with sickle-cell anemia (SCA) without stroke and HU treatment according to Transcranial Doppler (TCD).

Parameter	TCD1 (*N* = 73)	TCD2 (*N* = 20)	TCD3 (*N* = 13)	TCD2 + 3 (*N* = 33)	*p1*	*p*2
				
	50th (min-max)	50th (min-max)	50th (min-max)	50th (min-max)		
TAMMV	129 (90.10–167.00)	177.00 (171.00–196.00)	204.00 (201.00–231.00)	190.00 (171.00–231.00)	<0.0001**	**<0.0001^#^**
	**M ± SD**	**M ± SD**	**M ± SD**	**M ± SD**		
**Hemoglobin**						
HbF, %	11.85 ± 7.53	10.11 ± 4.71	7.55 ± 2.61	9.10 ± 4.16	0.2413**	0.2175**^##^**
HbS, %	83.31 ± 7.99	84.14 ± 5.10	85.95 ± 3.52	84.87 ± 4.54	0.7242**	0.7928**^##^**
**Hemolysis**						
RBC, ×10^6^/mL	3.34 ± 0.92	2.75 ± 0.36	2.82 ± 0.50	2.78 ± 0.41	0.0241**	**0.0063^##^**
Hemoglobin, g/dL	9.23 ± 1.73	8.44 ± 0.95	8.37 ± 0.72	8.42 ± 0.86	0.1022**	**0.0330^##^**
Hematocrit, %	27.27 ± 5.16	24.71 ± 3.12	24.44 ± 2.19	24.61 ± 2.77	0.0462**	**0.0135^##^**
Reticulocyte, %	7.07 ± 3.13	8.19 ± 2.02	8.14 ± 1.39	8.17 ± 1.78	0.2145**	0.0793**^##^**
Reticulocytes, /mL	218,813 ± 90,569	224,945 ± 70,502	232,993 ± 66,571	228,061 ± 67,993	0.8521*	0.6136**^#^**
MCV, fL	83.36 ± 9.74	90.29 ± 6.99	87.89 ± 9.52	89.39 ± 9.74	0.0091*	**0.0028^#^**
MCH, pg	28.44 ± 3.80	30.90 ± 2.89	30.24 ± 4.24	30.66 ± 3.40	0.0201*	**0.0058^#^**
MCHC, %	34.09 ± 1.81	34.20 ± 1.57	34.25 ± 1.70	34.22 ± 1.59	0.9350*	0.7182**^#^**
RDW, %	19.63 ± 3.47	20.51 ± 2.01	21.52 ± 2.55	20.89 ± 2.24	0.0741**	**0.0361^##^**
Haptoglobin, mg/dL	13.06 ± 29.71	5.83 ± 0.00	5.83 ± 0.00	5.83 ± 0.00	0.3268**	0.1347**^##^**
**Leukocytes**						
WBC, /mL	12,136 ± 4,448	12,870 ± 5,180	15,917 ± 3,051	14,012 ± 4,690	0.0291*	0.0554**^#^**
Lymphocyte, /mL	4,838 ± 2,713	5,586 ± 3,064	7,749 ± 2,381	6,397 ± 2,983	0.0040*	**0.0107^#^**
Eosinophil, /mL	881 ± 846	1,119 ± 1,124	1,198 ± 908	1,149 ± 1,034	0.3837*	0.1715**^#^**
Neutrophil, /mL	5,145 ± 2,388	5,109 ± 2,769	5,172 ± 1,323	5,133 ± 2,307	0.8772**	0.9807**^#^**
Monocyte, /mL	998 ± 514	820 ± 550	1,404 ± 548	1,039 ± 612	0.0111*	0.7247**^#^**
Basophil, /mL	97 ± 113	92 ± 87	156 ± 130	116 ± 108	0.2127*	0.4400**^#^**
**Platelets**						
Platelet, ×10^3^/mL	368 ± 134	377 ± 125	426 ± 112	396 ± 121	0.3585*	0.3204**^#^**
MPV, fL	6.92 ± 1.75	6.39 ± 1.24	6.12 ± 1.30	6.29 ± 1.25	0.1819*	0.0874**^##^**
**Hemolytic plus hepatic**						
Total bilirubin, mg/dL	1.99 ± 1.25	2.65 ± 1.59	2.43 ± 0.98	2.56 ± 1.36	0.1104*	**0.0402^#^**
Direct bilirubin, mg/dL	0.42 ± 0.14	0.45 ± 0.15	0.39 ± 0.13	0.43 ± 0.14	0.4906*	0.9575**^#^**
Indirect bilirubin, mg/dL	1.56 ± 1.21	2.19 ± 1.54	2.04 ± 0.96	2.13 ± 1.32	0.1016*	**0.0344^#^**
Lactate dehydrogenase, U/L	1076.30 ± 441	1339.26 ± 500.22	1306.15 ± 595.01	1325.81 ± 531.61	0.0509*	**0.0147^#^**
Iron serum, mcg/dL	82.81 ± 26.78	84.54 ± 33.82	93.44 ± 31.46	84.11 ± 32.41	0.9718*	0.8303**^#^**
Aspartate aminotransferase, U/L	45.17 ± 14.12	56.20 ± 17.16	55.08 ± 14.93	55.76 ± 16.09	0.0043*	**0.0009^#^**
**Lipids and glucose**						
Total cholesterol, mg/dL	129.37 ± 25.29	128.05 ± 22.85	130.15 ± 17.62	128.88 ± 20.68	0.9658*	0.9232**^#^**
HDL-C, mg/dL	41.98 ± 12.84	35.05 ± 7.77	37.46 ± 12.67	36.00 ± 9.87	0.0591**	**0.0197^#^**
LDL-C, mg/dL	69.65 ± 22.73	77.28 ± 19.71	72.57 ± 15.58	75.42 ± 18.09	0.3691*	0.2023**^#^**
Triglycerides, mg/dL	88.65 ± 34.77	78.10 ± 27.32	100.61 ± 42.93	86.97 ± 35.49	0.1889*	0.8204**^#^**
Glucose, mg/dL	68.15 ± 14.74	75.10 ± 14.22	78.23 ± 16.53	76.33 ± 14.99	0.0314*	**0.0102^#^**
**Renal**						
Urea, mg/dL	17.42 ± 5.70	18.45 ± 4.69	14.54 ± 2.70	16.91 ± 4.43	0.0628**	0.6487**^#^**
Creatinine, mg/dL	0.42 ± 0.11	0.40 ± 0.09	0.38 ± 0.07	0.39 ± 0.09	0.3486*	0.1920**^#^**
**Hepatic**						
Alanine aminotransferase, U/L	17.30 ± 9.87	22.95 ± 10.57	19.92 ± 10.28	21.76 ± 10.40	0.0823*	**0.0380^#^**
Total protein, g/dL	7.67 ± 0.77	7.53 ± 0.74	7.43 ± 0.56	7.49 ± 0.67	0.4649*	0.2377**^#^**
Albumin, g/dL	4.40 ± 0.61	4.39 ± 0.27	4.36 ± 0.30	4.38 ± 0.28	0.7726**	0.4756**^##^**
Globulin, g/dL	3.20 ± 0.76	3.12 ± 0.76	3.07 ± 0.71	3.10 ± 0.73	0.8139*	0.5432**^#^**
**Inflammatory**						
Ferritin, ng/dL	259.57 ± 557.97	277.07 ± 236.82	246.43 ± 156.19	264.62 ± 205.53	0.1829**	0.0666**^##^**
C-reactive protein, mg/L	17.12 ± 36.10	4.41 ± 4.39	5.58 ± 3.14	4.95 ± 3.83	0.2065**	0.3047**^##^**
Alpha 1 antitrypsin, mg/dL	133.61 ± 39.26	172.63 ± 56.51	143.42 ± 30.14	160.54 ± 48.95	0.0071*	**0.0097^#^**

TCD, Transcranial Doppler, TAMMV, Time-averaged maximum mean velocity; RBC, red blood cell; MCH, mean corpuscular hemoglobin; MCV, mean corpuscular volume; MCHC, mean corpuscular hemoglobin concentration; HbS, variant S hemoglobin; HbF, Fetal hemoglobin; RDW, red cell distribution width; HDL-C, high-density lipoprotein cholesterol; LDL-C, low-density lipoprotein cholesterol; WBC, white blood cell; MPV, mean platelet volume; M, mean; SD, standard deviation; p1, TCD1 vs. TCD2 vs. TCD3; p2, TCD1 vs. TCD2 + 3.

*ANOVA test, **Kruskal Wallis test, ^#^Independent T-test, ^##^Mann Whitney U test. 50th: 50th percentile, M: mean, SD: standard deviation, p1: p-value between TCD1 vs TCD2 vs TCD3, and p2: p-value between TCD1 vs TCD2+3; significant p-values are highlighted in bold.

According to the findings presented in Table 1, which showed similar or elevated values of some parameters in the TCD2 and TCD3 groups, we decided to combine these two groups and compare their laboratory parameters with those in the TCD1 group. Results showed that patients with non-normal TCD (TCD2 + TCD3) had significantly reduced RBC, hemoglobin, and hematocrit, as well as increased MCV, MCH, RDW total, and indirect bilirubin, LDH, AST, and ALT, when compared with those with normal TCD (TCD1). Moreover, they presented increased lymphocyte counts, glucose, and AAT levels, as well as a decrease in HDL-C levels (*p* < 0.05).

### Correlations between time-averaged maximum mean velocity and laboratory parameters

**FIGURE 2:**
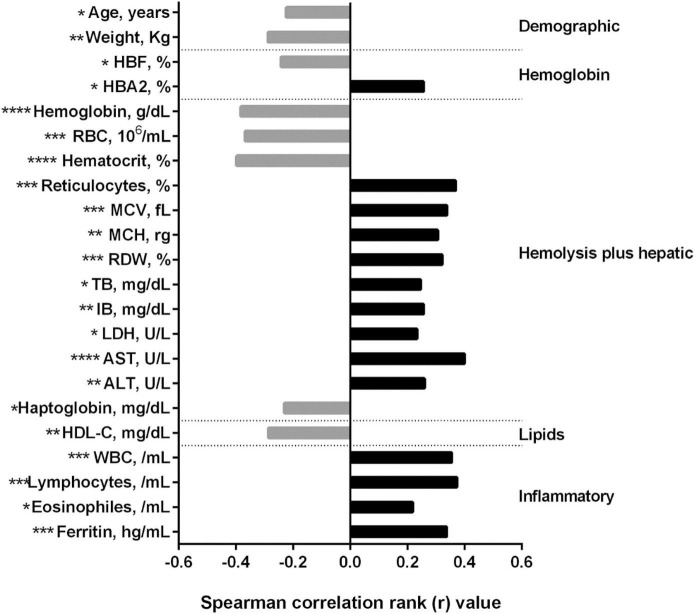
Correlations between TAMMV and laboratory markers in SCA children. Data were obtained using the Spearman correlation rank test. Bars represent the strength of correlation (r values). Gray and black bars indicate a negative and positive correlation, respectively. TAMMV, time-averaged maximum mean velocity; HBF, Fetal hemoglobin; HBA2, normal hemoglobin A2; RBC, red blood cells; MCV, mean corpuscular volume; MCH, mean corpuscular hemoglobin; RDW, red cell distribution width; TB, total bilirubin; IB, indirect bilirubin; LDH, lactate dehydrogenase; AST, aspartate aminotransferase; ALT, alanine aminotransferase; HDL-C, high-density lipoprotein cholesterol; WBC, white blood cells. *p < 0.05, **p < 0.01, ***p < 0.001 and ****p < 0.0001.

Figure 2 shows the results of the correlation analyses. The analysis of the demographic data showed a negative correlation between TAMMV and age, as well as weight (*p* < 0.05). TAMMV presented a negative correlation with HbF and a positive correlation with HbA2 (*p* < 0.05). Regarding the hemolysis and hepatic profile, TAMMV was negatively correlated with hemoglobin, RBC, hematocrit, and haptoglobin, as well as positively correlated with reticulocytes, MCV, MCH, RDW, total and indirect bilirubin, LDH, AST and ALT (*p* < 0.05). An analysis of the lipid profile showed a negative correlation between TAMMV and HDL-C (*p* < 0.05). Moreover, we also observed a positive correlation between TAMMV and inflammatory profile (WBC, lymphocyte and eosinophil counts, and ferritin levels).

## Discussion

In this study, we investigated the association between laboratory biomarkers and cerebral blood flow velocity in SCA children from Bahia State in Brazil. Reports show higher stroke incidence in young children, specifically between 2 and 5 years old (8). During our study, any patient had no type of stroke. This can be explained as most of our patients were over 5 years old. It could also be an underestimate of silent strokes, given that our patients did not have access to magnetic resonance imaging MRI or MRA, which are more sensitive procedures to detect silent infarction (SCI) (25–27). As expected, the analysis of the TAMMV values between the three different groups investigated showed significantly lower TAMMV values in the TCD1 group compared to those with TCD2 or TCD3. This finding corroborates the results of previous studies (8, 28). The cerebral hemodynamics in SCA is quite complex and has been studied to better understand how it influences the risk of stroke and to consider appropriate therapeutic strategies (29, 30). Elevated cerebral blood flow velocity (CBFV), in patients with SCA, has been related to severe anemia and cerebral vasodilatation (31). Moreover, in the context of cardiovascular disease, anemia is associated with the occurrence of cerebrovascular events (32, 33). Hypoxic conditions are associated with increased CBF velocity and a dilation of the brain vasculature to maintain tissue oxygenation (30, 34).

In this study, laboratory biomarker analysis showed that children with non-normal TCD (TCD2 and/or TCD3 group) had significantly reduced RBC, hemoglobin, and hematocrit when compared with the group with normal TCD (TCD1). These findings corroborate the results from Adams et al., who also demonstrated the association between the reduction in these hemolytic markers and the increase in CBFV (28). Reports suggested anemia as an important risk factor for stroke event occurrence (8, 35, 36). Indeed, the compensatory response to anemia in SCA may lead to an increase in CBFV due to vasodilatation of the cerebral vasculature (29, 37–39). Chan and Ganasekaran explained that anemia can cause alterations in cerebral blood vessels, and change oxygenation in the brain, thereby increasing stroke risk (40). Moreover, children with non-normal TCD exhibited higher MCH, MCV, and RDW, which may be explained by the severe anemia in this group, when compared with those with normal TCD. This finding confirms a previous report, suggesting the lower MCH as a protective marker regarding stroke occurrence (41). When higher MCH is predictive of stroke occurrence, higher MCV was reported as a death risk marker post-stroke, in the short term (42). Regarding RDW, its increase is known to be a predictor of poor stroke outcomes (43, 44). Previous studies conducted in the Brazilian population showed associations between reductions in MCH and MCV levels and alpha thalassemia prevalence (45–47). α-thalassemia was shown to present a protective effect against the risk of abnormal TCD in Nigerian children with SCA (48). Moreover, studies conducted in HbSC and HbSS patients did not observe any association between α-thalassemia and TAMMV (49, 50).

Children with non-normal TCD also had a significant increase in AST levels compared with to those with normal TCD, which may be a risk factor for stroke occurrence as suggested by a previous study (51). Moreover, TCD3 group presented significantly elevated total and indirect bilirubin, ALT, and LDH levels, when compared with the TCD1 group, corroborating the previous data, suggesting a more exacerbated hemolytic profile in the TCD3 group than TCD1 group. Moreover, these data corroborate findings from the study by O’Driscoll et al., which also found a positive correlation between LDH and TAMMV (51).

Analysis of leukocyte and inflammatory profiles showed significantly increased counts of WBC, lymphocytes, and monocytes, and AAT levels in the TCD3 group, when compared with those in TCD1. These findings corroborate a previous study, which found a positive correlation between leukocytes and TAMMV (52). During the cerebral ischemic mechanism, activated leukocytes are highly implicated and are considered a predictor of the outcome (53). Moreover, children with non-normal TCD present increased lymphocyte counts in compared with children with normal TCD. The increase in these inflammatory biomarkers, specifically in the TCD3 group when compared to the TCD1 group, besides to confirming the association of these biomarkers with stroke risk, suggests an association between recruitment of activated-inflammatory cells and acute stroke occurrence (43, 54). Moreover, studies found elevated leukocyte counts in patients who suffered a stroke when compared with controls (43, 53). Indeed, the exacerbated inflammatory condition, involving recruitment, activation, and adhesion of these inflammatory cells to the cerebral vascular endothelial is considered one of the main mechanisms underlying the increase in blood velocity and consequently stroke events (35). Interestingly, we observed an increase in glucose levels in the TCD3 group when compared to TCD1 group. Corroborating this finding, a previous study also demonstrated elevated glucose levels in patients with SCD in the period before and after acute stroke events (55). Although the relationship between glucose and stroke events is unclear, hyperglycemia is known to worsen stroke outcomes due to its association with increases in brain injury (55). Moreover, studies have shown that hyperglycemia increases coagulation, by promoting thrombin production (56), as well as brain tissue damage (57).

Correlation analyses showed a negative correlation between TAMMV and age, corroborating the findings of previous studies, where the TAMMV decreases as the children get older (52, 58). A Nigerian study reported that CBFV peaks during the first 6 years and tends to decline with advancing age (59). Generally, the decline in CBFV with age may be explained by changes in cerebrovascular hemodynamics linked to less metabolic demands (60). According to our findings, children with elevated weight are susceptible to an increase in TAMMV, consequently stroke risk. However, this finding deserves to be better investigated. A study in Jamaica showed a similar correlation but not sustained when they associated TAMV with BMI z-score or height-age z-score (61). We also observed a negative correlation between TAMMV and HbF and, its positive correlation with HbA2. This finding corroborates the association between higher HbF levels and a reduction in the occurrence of silent cerebral infarctions and overt strokes (62, 63). Analyses also demonstrated negative correlations between TAMMV and hemoglobin, RBC, and hematocrit, as well as positive correlations between TAMMV and reticulocytes, MCV, MCH, RDW, total and indirect bilirubin, LDH, AST, and ALT. Previous studies found a negative correlation between TAMMV and hematocrit levels (64, 65); (66, 67) reported that higher serum LDH levels may predict poor outcomes of Aneurysmal Subarachnoid Hemorrhage, while other studies found positive correlation between LDH and TAMM velocity.

Some studies have related the permeability of the Blood-brain barrier by the free bilirubin (BF), then, mechanisms of inflammatory mediators released by astrocytes activated by unconjugated bilirubin (UCB), may contribute to cerebral vasodilation. Also, an increase in CBFV was observed in neonates with hyperbilirubinemia (68, 69).

Serum ALT levels showed significant positive correlations with CBFV in the superior frontal gyrus and negative correlations with CBFV in the middle occipital gyrus, angular gyrus, precuneus, and middle temporal gyrus. But no association with AST (70).

Results also demonstrated a positive correlation between TAMMV and inflammatory biomarkers (WBC, lymphocyte and eosinophil counts, and ferritin levels). Serum ferritin has also been described as a risk factor for stroke in post-menopausal women (71). A previous study demonstrated that increased ferritin levels are correlated with stroke severity and the size of the lesion (72). Furthermore, elevated ferritin levels could be associated with a chronic inflammatory state and chronic hemolytic condition as observed in our population (50). The inflammatory state is evidenced by increases in liver function biomarkers, such as AST, which, in this study, was positively correlated with TAMMV. Regarding the lipid profile, we observed a negative correlation between TAMMV and HDL-C. This finding corroborates a previous study, which suggests an association between higher HDL-C levels and reduced stroke risk (73), which can be explained by the beneficial effects of HDL-C on endothelial function. Previous studies observed significantly increased cerebral CO_2_ reactivity with increasing levels of serum HDL-C and decreased cerebral CO_2_ reactivity with an increasing cholesterol/HDL-C ratio (74, 75).

## Conclusion

Results of this study highlight the importance of the investigation of hemolytic and inflammatory biomarkers for monitoring the clinical outcome of SCA pediatric patients, to avoid acute or chronic stroke. Moreover, glucose and HDL-C appear to be useful for predicting higher TAMMV i.e., stroke risk. Further studies are needed to understand this relationship and establish a cutoff value, which may be used to identify with precision, in association with hemolytic and inflammatory biomarkers, patients with stroke risk.

## Data availability statement

The original contributions presented in this study are included in the article/Supplementary material, further inquiries can be directed to the corresponding author.

## Ethics statement

This study received approval from the Institutional Review Board of the HUPES (protocol number: 287.768/2013). Written informed consent to participate in this study was provided by the participants or their legal guardian/next of kin.

## Author contributions

CA and MG conceived and designed the study. CA, SY, RS, SS, JS, TP, MA, and VM collected the samples. CA, SY, RS, SS, DZ, and JS performed the experiments. CA and SY analyzed the data. CA wrote the manuscript. CV, IPL, and IML followed the patients. CV performed the TCD examine. MG supervised the study and critically revised the manuscript. All authors revised and approved the manuscript.
