# Supplementary material for: Association of laboratory markers and cerebral blood flow among sickle cell anemia children
Source: Front Pediatr. 2022 Aug 26;10:914466. doi: 10.3389/fped.2022.914466 (PMC9458965; doi:10.3389/fped.2022.914466)
Supplement: Supplementary file 1 [file Presentation_1.pptx]

## Slide 1
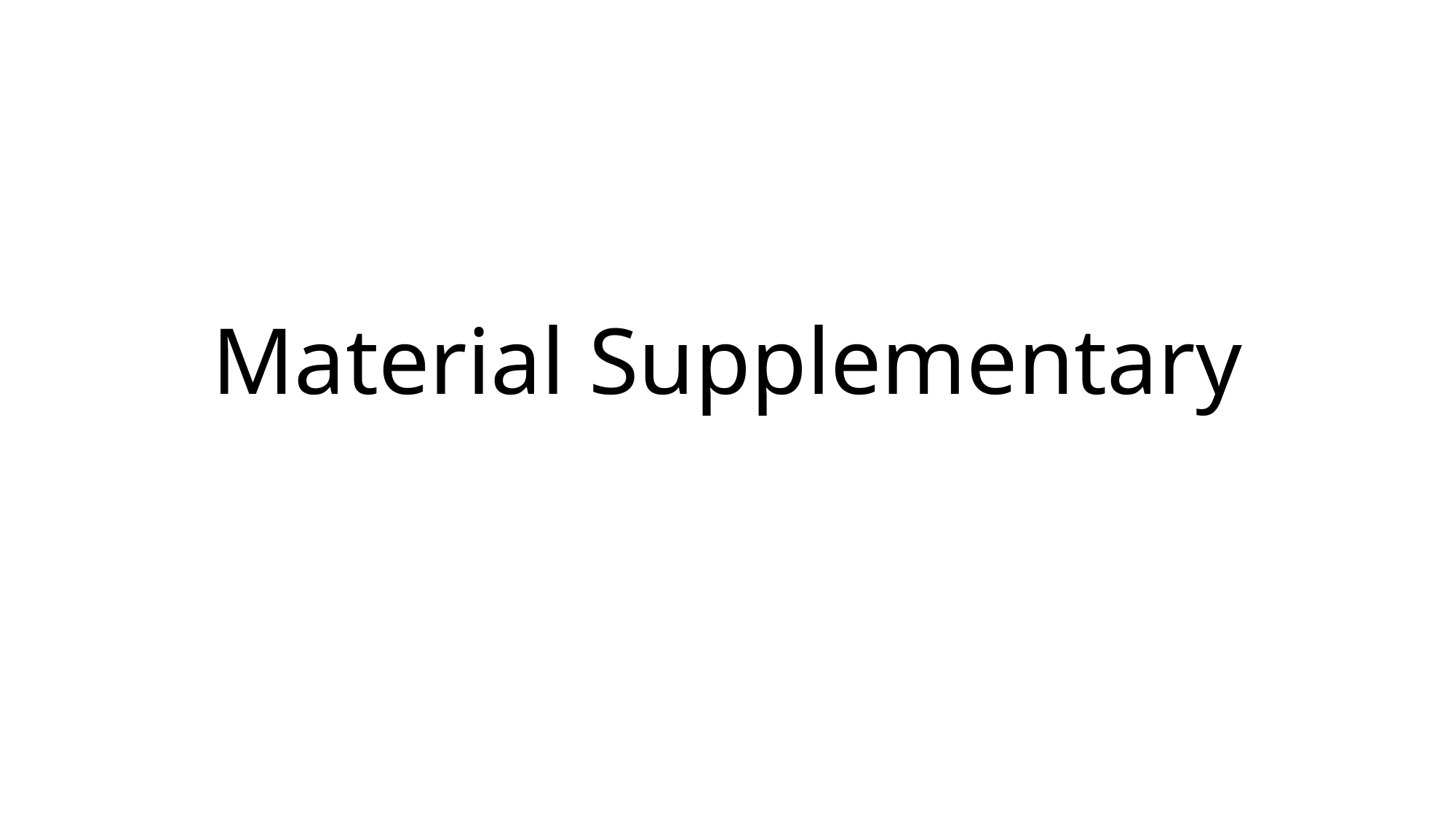

# Material Supplementary

## Slide 2
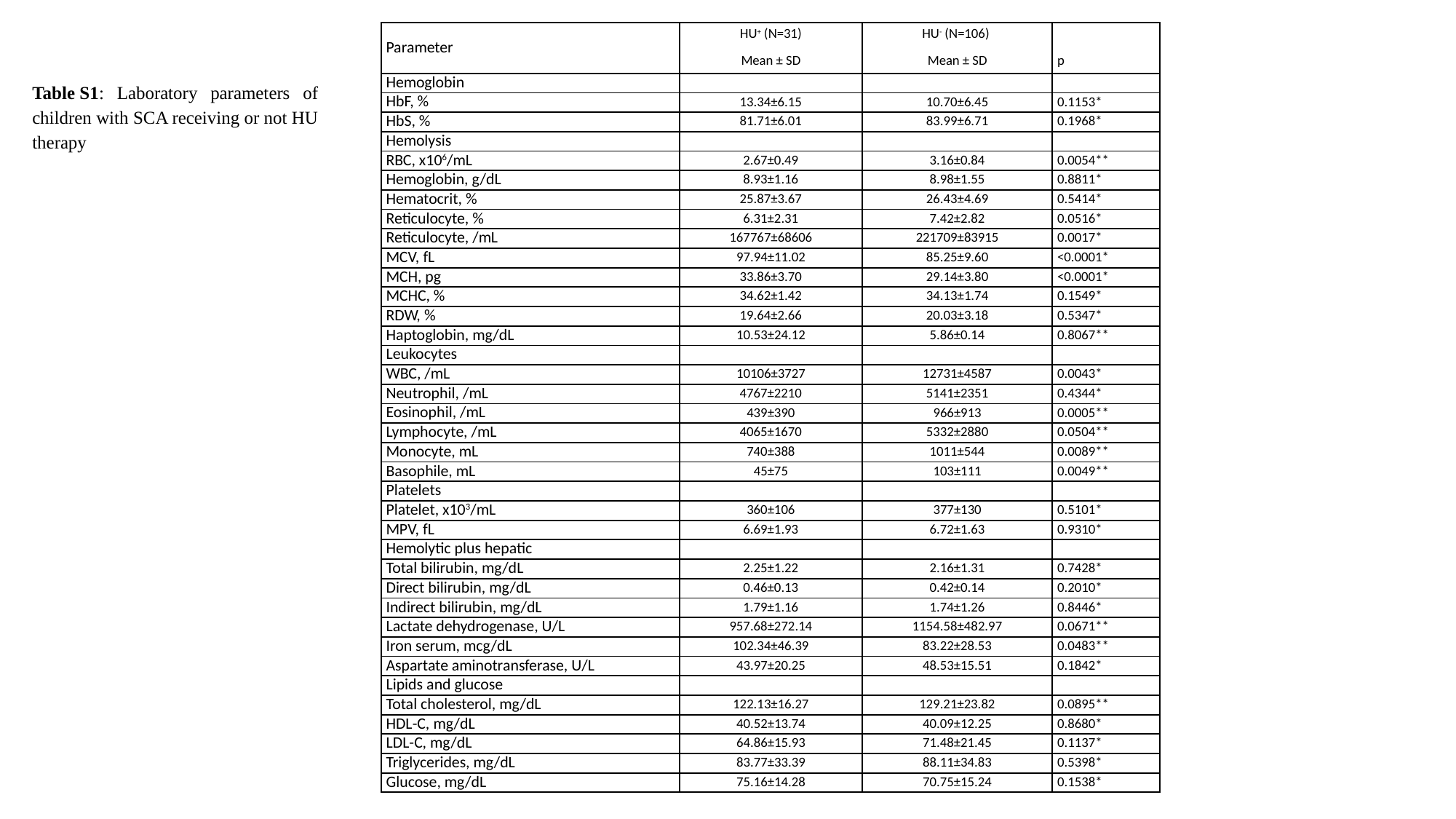

| Parameter | HU+ (N=31) Mean ± SD | HU- (N=106) Mean ± SD | p |
| --- | --- | --- | --- |
| Hemoglobin | | | |
| HbF, % | 13.34±6.15 | 10.70±6.45 | 0.1153\* |
| HbS, % | 81.71±6.01 | 83.99±6.71 | 0.1968\* |
| Hemolysis | | | |
| RBC, x106/mL | 2.67±0.49 | 3.16±0.84 | 0.0054\*\* |
| Hemoglobin, g/dL | 8.93±1.16 | 8.98±1.55 | 0.8811\* |
| Hematocrit, % | 25.87±3.67 | 26.43±4.69 | 0.5414\* |
| Reticulocyte, % | 6.31±2.31 | 7.42±2.82 | 0.0516\* |
| Reticulocyte, /mL | 167767±68606 | 221709±83915 | 0.0017\* |
| MCV, fL | 97.94±11.02 | 85.25±9.60 | <0.0001\* |
| MCH, pg | 33.86±3.70 | 29.14±3.80 | <0.0001\* |
| MCHC, % | 34.62±1.42 | 34.13±1.74 | 0.1549\* |
| RDW, % | 19.64±2.66 | 20.03±3.18 | 0.5347\* |
| Haptoglobin, mg/dL | 10.53±24.12 | 5.86±0.14 | 0.8067\*\* |
| Leukocytes | | | |
| WBC, /mL | 10106±3727 | 12731±4587 | 0.0043\* |
| Neutrophil, /mL | 4767±2210 | 5141±2351 | 0.4344\* |
| Eosinophil, /mL | 439±390 | 966±913 | 0.0005\*\* |
| Lymphocyte, /mL | 4065±1670 | 5332±2880 | 0.0504\*\* |
| Monocyte, mL | 740±388 | 1011±544 | 0.0089\*\* |
| Basophile, mL | 45±75 | 103±111 | 0.0049\*\* |
| Platelets | | | |
| Platelet, x103/mL | 360±106 | 377±130 | 0.5101\* |
| MPV, fL | 6.69±1.93 | 6.72±1.63 | 0.9310\* |
| Hemolytic plus hepatic | | | |
| Total bilirubin, mg/dL | 2.25±1.22 | 2.16±1.31 | 0.7428\* |
| Direct bilirubin, mg/dL | 0.46±0.13 | 0.42±0.14 | 0.2010\* |
| Indirect bilirubin, mg/dL | 1.79±1.16 | 1.74±1.26 | 0.8446\* |
| Lactate dehydrogenase, U/L | 957.68±272.14 | 1154.58±482.97 | 0.0671\*\* |
| Iron serum, mcg/dL | 102.34±46.39 | 83.22±28.53 | 0.0483\*\* |
| Aspartate aminotransferase, U/L | 43.97±20.25 | 48.53±15.51 | 0.1842\* |
| Lipids and glucose | | | |
| Total cholesterol, mg/dL | 122.13±16.27 | 129.21±23.82 | 0.0895\*\* |
| HDL-C, mg/dL | 40.52±13.74 | 40.09±12.25 | 0.8680\* |
| LDL-C, mg/dL | 64.86±15.93 | 71.48±21.45 | 0.1137\* |
| Triglycerides, mg/dL | 83.77±33.39 | 88.11±34.83 | 0.5398\* |
| Glucose, mg/dL | 75.16±14.28 | 70.75±15.24 | 0.1538\* |
Table S1: Laboratory parameters of children with SCA receiving or not HU therapy

## Slide 3
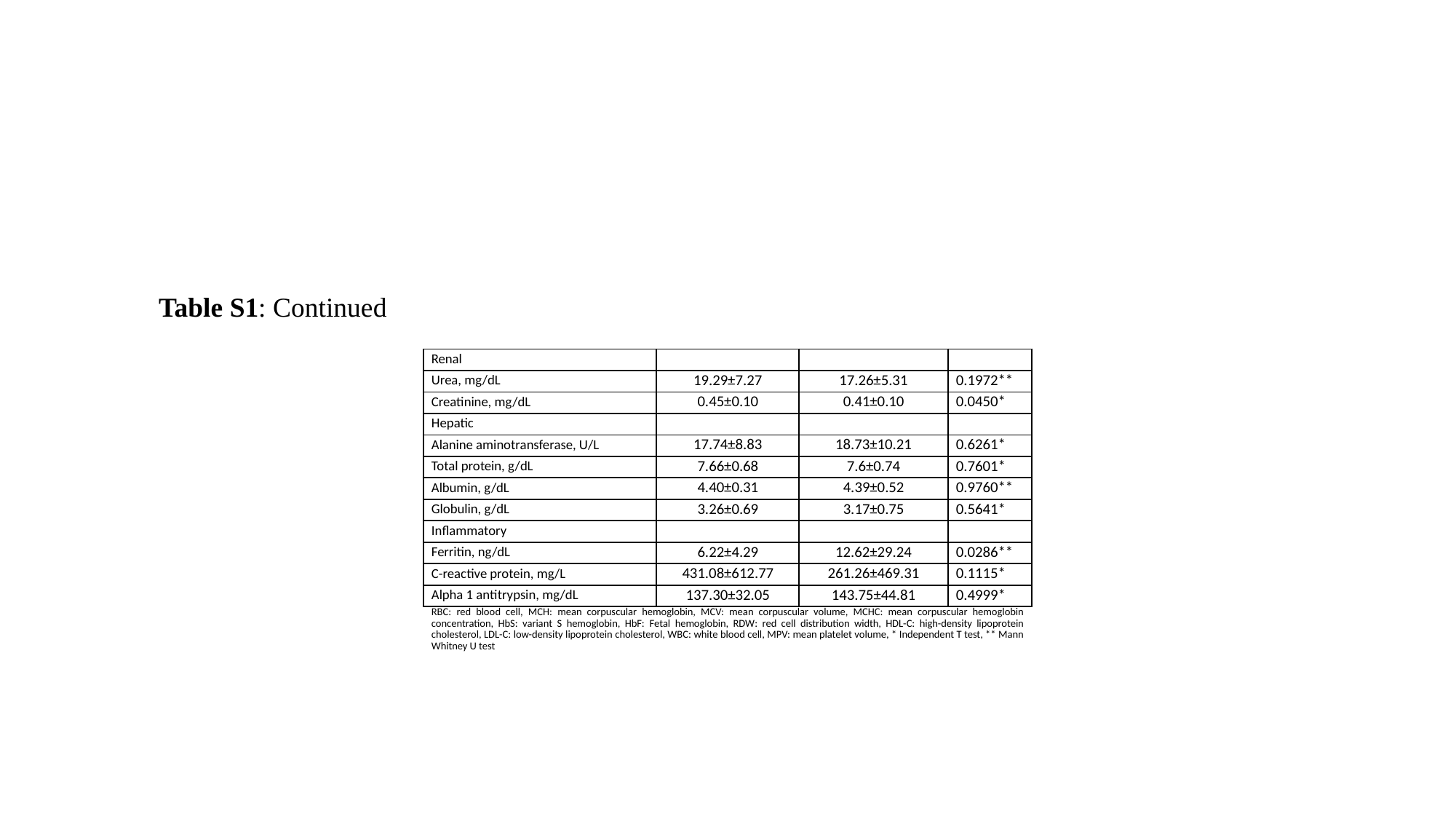

Table S1: Continued
| Renal | | | |
| --- | --- | --- | --- |
| Urea, mg/dL | 19.29±7.27 | 17.26±5.31 | 0.1972\*\* |
| Creatinine, mg/dL | 0.45±0.10 | 0.41±0.10 | 0.0450\* |
| Hepatic | | | |
| Alanine aminotransferase, U/L | 17.74±8.83 | 18.73±10.21 | 0.6261\* |
| Total protein, g/dL | 7.66±0.68 | 7.6±0.74 | 0.7601\* |
| Albumin, g/dL | 4.40±0.31 | 4.39±0.52 | 0.9760\*\* |
| Globulin, g/dL | 3.26±0.69 | 3.17±0.75 | 0.5641\* |
| Inflammatory | | | |
| Ferritin, ng/dL | 6.22±4.29 | 12.62±29.24 | 0.0286\*\* |
| C-reactive protein, mg/L | 431.08±612.77 | 261.26±469.31 | 0.1115\* |
| Alpha 1 antitrypsin, mg/dL | 137.30±32.05 | 143.75±44.81 | 0.4999\* |
| RBC: red blood cell, MCH: mean corpuscular hemoglobin, MCV: mean corpuscular volume, MCHC: mean corpuscular hemoglobin concentration, HbS: variant S hemoglobin, HbF: Fetal hemoglobin, RDW: red cell distribution width, HDL-C: high-density lipoprotein cholesterol, LDL-C: low-density lipoprotein cholesterol, WBC: white blood cell, MPV: mean platelet volume, \* Independent T test, \*\* Mann Whitney U test | | | |

## Slide 4
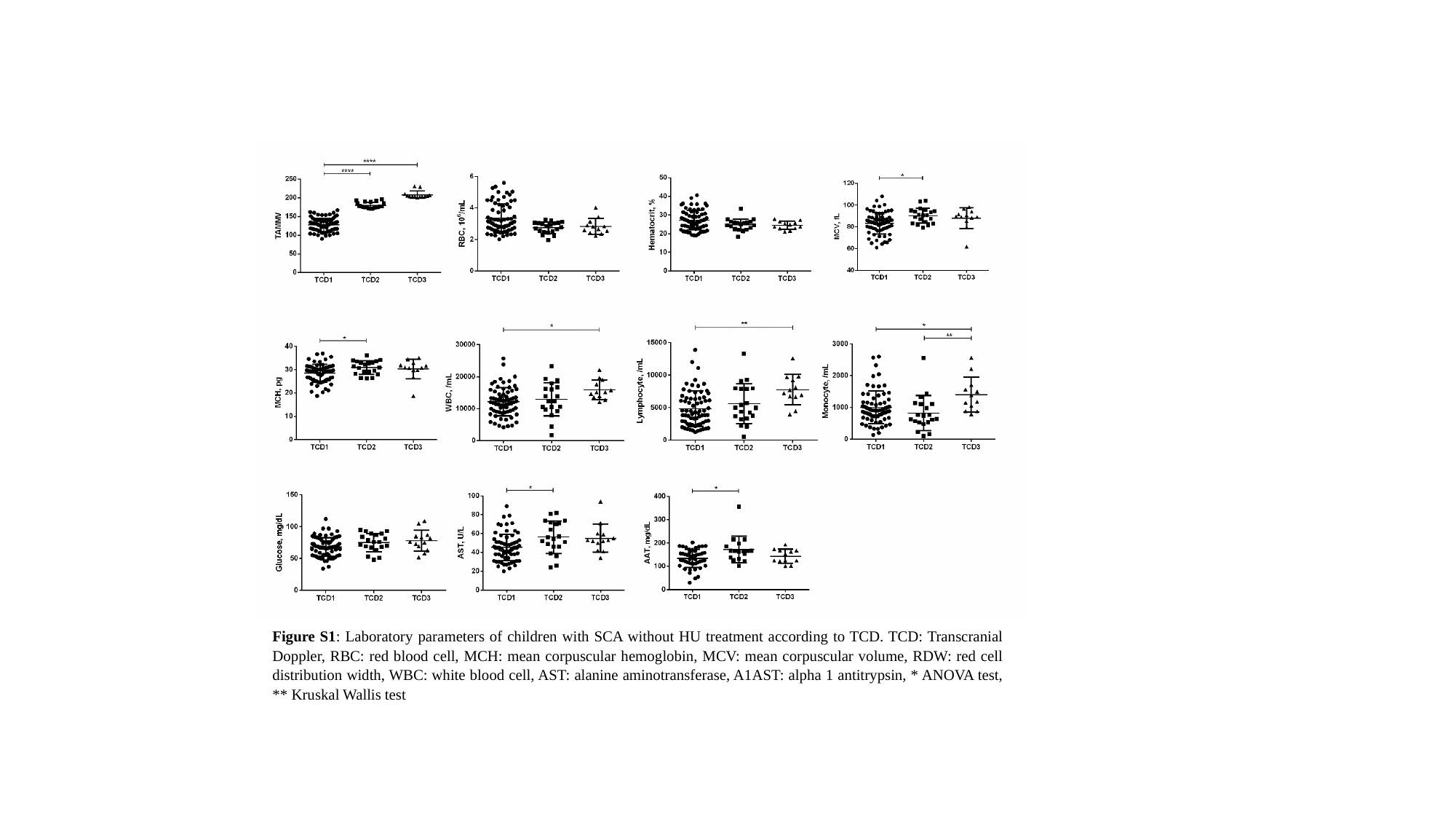

Figure S1: Laboratory parameters of children with SCA without HU treatment according to TCD. TCD: Transcranial Doppler, RBC: red blood cell, MCH: mean corpuscular hemoglobin, MCV: mean corpuscular volume, RDW: red cell distribution width, WBC: white blood cell, AST: alanine aminotransferase, A1AST: alpha 1 antitrypsin, * ANOVA test, ** Kruskal Wallis test
